# Supplementary material for: Landscape to microhabitat: Uncovering the multiscale complexity of native and exotic forests on Terceira Island (Azores, Portugal)
Source: PLoS One. 2025 Jun 16;20(6):e0326304. doi: 10.1371/journal.pone.0326304 (PMC12169593; doi:10.1371/journal.pone.0326304)
Supplement: S1 Data — (ZIP) [file pone.0326304.s001.zip › Supp_mat (data)/Table S1 - Variables description.docx]

***Table S1.*** ***Description of the 49 variables used in the NMDS analysis****.*

| **Variable abbreviation** | **Description** | **Data source** | **Group** |
| --- | --- | --- | --- |
| agriculture_prop | Proportion of agricultural land use within a 500 m radius of each plot. | Landscape analysis | Spatial trait |
| naturalveg_prop | Proportion of natural vegetation within a 500 m radius of each plot. | Landscape analysis | Spatial trait |
| openarea_prop | Proportion of open or barren land within a 500 m radius of each plot. | Landscape analysis | Spatial trait |
| pasture_prop | Proportion of pasture land use within a 500 m radius of each plot. | Landscape analysis | Spatial trait |
| secondaryforest_prop | Proportion of secondary forest land use within a 500 m radius of each plot. | Landscape analysis | Spatial trait |
| urban_prop | Proportion of urban land use within a 500 m radius of each plot. | Landscape analysis | Spatial trait |
| slope_max | Maximum terrain slope (°) within a 500 m radius. | Landscape analysis | Spatial trait |
| slope_mean | Mean terrain slope within each plot area within a 500 m radius. | Landscape analysis | Spatial trait |
| slope_min | Minimum terrain slope within a 500 m radius. | Landscape analysis | Spatial trait |
| slope_std | Standard deviation of terrain slope within a 500m radius. | Landscape analysis | Spatial trait |
| SRTM_elevation | Elevation of each plot, as derived from SRTM (Shuttle Radar Topography Mission) data. | Landscape analysis | Spatial trait |
| mean_FD | Mean fractal dimension. | Terrestrial laser scanning | Stand trait |
| SSCI | Structural Complexity Index of the canopy across plots after [(Ehbrecht et al. 2017)](https://www.zotero.org/google-docs/?jsrtpy). | Terrestrial laser scanning | Stand trait |
| canopy_openness | Percentage of canopy openness across plot areas. | Terrestrial laser scanning | Canopy trait |
| UCI_mean | Understory complexity index after [(Willim et al. 2019)](https://www.zotero.org/google-docs/?5y4ozl); The higher the value, the more structural elements were present between 0.8 and 1.8 meters above ground. | Terrestrial laser scanning | Understory Trait |
| ENL0D | Stand height (m) for the ground layer of vegetation. | Terrestrial laser scanning | Stand trait |
| ENL1D | Weighted effective number of layers following [(Ehbrecht et al. 2016)](https://www.zotero.org/google-docs/?QcrHbr). | Terrestrial laser scanning | Stand trait |
| ENL2D | Weighted effective number of layers following [(Ehbrecht et al. 2016)](https://www.zotero.org/google-docs/?7PQBKI). | Terrestrial laser scanning | Stand trait |
| foliage_height_diversity | Measure for the vertical layering and number of different vertical layers after [(MacArthur and MacArthur 1961)](https://www.zotero.org/google-docs/?mLKwSs). | Terrestrial laser scanning | Stand trait |
| vertical_evenness | Vertical evenness of vegetation distribution across vertical layers (1m thick). | Terrestrial laser scanning | Stand trait |
| roughness_avg | Average roughness of canopy surface within each plot. | UAV mapping | Canopy community trait |
| roughness_std | Standard deviation of canopy roughness. | UAV mapping | Canopy community trait |
| basal_area_endemic | Basal area (m²/ha) of trees classified as endemic species. | Vegetation survey | Canopy community trait |
| basal_area_introduced | Basal area of trees classified as introduced species. | Vegetation survey | Canopy community trait |
| basal_area_invasive | Basal area of trees classified as invasive species. | Vegetation survey | Canopy community trait |
| basal_area_native | Basal area of trees classified as native species. | Vegetation survey | Canopy community trait |
| density_0_endemic | Density (shoots/m²) of endemic species in the ground layer. | Vegetation survey | Ground community trait |
| density_0_introduced | Density of introduced species in the ground layer. | Vegetation survey | Ground community trait |
| density_0_invasive | Density of invasive species in the ground layer. | Vegetation survey | Ground community trait |
| density_0_native | Density of native species in the ground layer. | Vegetation survey | Ground community trait |
| density_1_endemic | Density of endemic species in the understory layer. | Vegetation survey | Understory community trait |
| density_1_introduced | Density of introduced species in the understory layer. | Vegetation survey | Understory community trait |
| density_1_invasive | Density of invasive species in the understory layer. | Vegetation survey | Understory community trait |
| density_1_native | Density of native species in the understory layer. | Vegetation survey | Understory community trait |
| density_2_endemic | Density of endemic species in the canopy layer. | Vegetation survey | Canopy community trait |
| density_2_introduced | Density of introduced species in the canopy layer. | Vegetation survey | Canopy community trait |
| density_2_invasive | Density of invasive species in the canopy layer. | Vegetation survey | Canopy community trait |
| density_2_native | Density of native species in the canopy layer. | Vegetation survey | Canopy community trait |
| 0_endemic | Number of endemic species in the ground layer. | Vegetation survey | Ground community trait |
| 0_introduced | Number of introduced species in the ground layer. | Vegetation survey | Ground community trait |
| 0_native | Number of native species in the ground layer. | Vegetation survey | Ground community trait |
| 1_endemic | Number of endemic species in the understory layer. | Vegetation survey | Understory community trait |
| 1_invasive | Number of invasive species in the understory layer. | Vegetation survey | Understory community trait |
| 1_native | Number of native species in the understory layer. | Vegetation survey | Understory community trait |
| 2_endemic | Number of endemic species in the canopy layer. | Vegetation survey | Canopy community trait |
| 2_introduced | Number of introduced species in the canopy layer. | Vegetation survey | Canopy community trait |
| 2_invasive | Number of invasive species in the canopy layer. | Vegetation survey | Canopy community trait |
| 2_native | Number of native species in the canopy layer. | Vegetation survey | Canopy community trait |
| H0_VP | Total number of vascular plant species. | Vegetation survey | Stand trait |

*Variables considered include vegetation attributes, structural complexity metrics, and landscape characteristics collected across native and exotic forest plots on Terceira Island. Variables are grouped according to their ecological meaning, namely: Spatial trait (data about the spatial geographical of the plots), Stand trait (data about the stand structure and global plant composition), Canopy community trait (data about the composition and structure of the canopy layer), Understory community trait (data about the composition and structure of the understory layer), Ground community trait (data about the composition and structure of the ground layer).*
