# Supplementary material for: Landscape to microhabitat: Uncovering the multiscale complexity of native and exotic forests on Terceira Island (Azores, Portugal)
Source: PLoS One. 2025 Jun 16;20(6):e0326304. doi: 10.1371/journal.pone.0326304 (PMC12169593; doi:10.1371/journal.pone.0326304)
Supplement: Attachment — Submitted filename: Comments on PONE_D_24_59937_Landscape to Microhabitat.pdf [file pone.0326304.s002.pdf]

Abstract *… By analysing landscape, habitat, and microhabitat traits, we found that native forests are situated in less accessible terrains….* rephrase, the location of native forests is not a trait, or derived from the fact that they are native, just the opposite, they are the remains of a former native forest covering all the locations.

Abstract *… homogeneous structural complexity derived from endemic vascular plant species….* I do not agree with "derived", it should be "and", adding enough exotic species or letting succession into the non-native forest will eventually result in complexity, complexity does not derive from endemicity (see comments on the Results).

Abstract *...exotic forests, which are situated in human-modified landscapes*; Exotic woodlands are the human modified landscape, rephrase as "exotic forests, are the result of human actions, afforestation or introduction of invading tree plants.

Abstract *...The ground and canopy layers in exotic forests were more invaded by nonindigenous species, while the understory demonstrated greater resilience...* very confusing statement, first an absolute need to resolve the difference between afforested areas and invaded areas, then also to differential use and actions of both, usually afforested areas have periodical cleaning of the understory?

Abstract *...Our findings suggest a temporal progression of forest change, from pristine native to disturbed exotic stands.*; The authors have no follow up of the dynamics of the forest to support this statement, by contrary many references exist proving the Azores forest cover suffered a massive and historical change, the object of study are the results of the change, conclusions cannot be to prove the chosen model of exotic versus native exists, something that should be stated in the abstract as historical, but by contrary to explain the complexity, diversity etc of the 2 distinct "woodlands"

Abstract "*...These insights highlight the necessity for scale-specific conservation, prioritising native forests in remote areas and controlling invasive species in exotic forests to enhance ecological resilience*"; basically what the authors propose is to protect what already is protected not because is legally protected but because is as stated "in less accessible terrains" and to keep the exotic areas as exotic although this exotic are themselves invasive, this is an absurd

Introduction The structure of the introduction is inverted, the object of study should firstly be defined and then the first 3 paragraphs on the first page should follow.

Introduction *The Azores, one of the most remote archipelagos in the North Atlantic Ocean, is made up of nine islands where forests contain a mix of native and exotic species, shaped by both natural processes and six centuries of human influence. (Elias et al. 2016).* This or a modified version of this sentence should be the opening sentence of the Introduction, the phrase is however overly optimistic in the use of the concept of "Mosaic" or "Mix", the Azores have certainly less than 5% of Native forest cover and therefore a Mosaic of 5% versus 95% is not a mosaic. This needs to be clearly expressed by the authors on the introduction. Several studies published by one of the authors (Elias) can be used to state and support this key point to the readers.

Introduction *...consist primarily of broad-leaved evergreen trees*. There are according to Elias (2016), Dias et al. (2021), and others several types of vegetation including some that are not broad-lead. To characterize the broad-leaved evergreen trees as such without any further hint or name is insufficient; include also the references: Dias, Eduardo; Mendes, Cândida; Aguiar, Carlos (2021). Vegetação dos Açores. In Capelo, J.; Aguiar, C. (Eds.), Vegetação de Portugal. Lisboa: INCM, p. 155-179. ISBN 978-972-27-2879-9; Mucina, L.; Bültmann, H.; Dierßen, K.; Theurillat, J.-P.; Raus, T.; Carni, A.; Šumberová, K.; et al. "Vegetation of Europe: hierarchical floristic classification system of vascular plant, bryophyte, lichen, and algal communities". Applied Vegetation Science 19 (2016): 3-264; Fernández Prieto, J.C.; Aguiar, C.; Dias, E.; Fernández Prieto, J.A.; Aguiar, Carlos; Dias, Eduardo;

FernÃ¡ndez Prieto, JosÃ© Antonio. "Description of some new syntaxa from the Azores archipelago". International Journal of Geobotanical Research 2 1 (2012): 111-116. http://dx.doi.org/10.5616/ijgr120007

Introduction ...*many endemic taxa* Characterize "Many", or compare with other similar forests, however we lack the sense/definition of forest and therefore a way to compare with other similar forests. There is reference in this introduction characterizing the types of forest and giving proper biogeographical framework.

Introduction ...*As a result, the Azores's landscape is now a mosaic of native and exotic forests, each playing a different ecological role…* This is a bold statement and a conclusion, furthermore without references, see above the comment on the "Mosaic", do invading exotic plants play a role is this a political or a scientific statement?

Introduction ...*Native forests are crucial for maintaining the Azorean island's biodiversity, acting as refuges for endemic plants and animals (Lhoumeau and Borges 2023)* Well… native forests are native in fact.

Introduction *Finally, the vegetation data collected from surveys provides information on species composition and density across all layers. The data, gathered on Terceira Island, are used to address the following question: What are the defining characteristics of the vegetation structure of the various types of Azorean forests, with a particular focus on the native and exotic woodlands? By answering this question, this study aims to provide a detailed characterisation of Terceira's forests, offering insights into how both native and exotic forest types are integrated within the island's broader ecosystem.* The reader finds out that after all the study concerns one island out of nine islands, that needs to be addressed on the proposed title "Landscape to Microhabitat: Uncovering the Multiscale Complexity of Terceira Island forests (Azores, Portugal)" but see further comments below.

Material and Methods. Study Area and Site selection The second part of the first paragraph needs to be moved to the introduction (to a new and so far lacking characterization of the Terceira forests and habitats)

Material and Methods. Study Area and Site selection The sentence "the third largest of the nine islands in the archipelago (figure 1)." should be moved to the first paragraph

Material and Methods. Study Area and Site selection The sentence "which are characterised by broadleaved evergreen trees and are home to a variety of endemic plant species" is conclusive and derives from the results themselves should deleted.

Material and Methods. Study Area and Site selection What is meant by "*by exotic vegetation, primarily consisting of Pittosporum undulatum*", *Pittosporum undulatum* is an invasive plant species very well known, documented etc. therefore 1) there should be detailed information on this species on the introduction, including reference to many publications concerning the Azores/*Pittosporum undulatum* question; 2) *Pittosporum undulatum* is an Invasive tree species and should be delt as invasive; 3) if there are no other dominant exotic species referred on the study (such as *Cryptomeria japonica*, the dominant exotic tree in the Azores... then the title of the manuscript should be further adapted/changed to fit this much more simple comparison. One Island and Native (even this can be explicit) versus Pittosporum invaded areas.

Material and Methods. Study Area and Site selection There is a lacking explanation on the 18 plots choice, it cannot just be because they are already in use in something else. Are they bioclimaticaly homogeneous? Are they similar in use, in historical use? Etc. A table with all the plots and as many characteristics as possible should be included, namely altitude, slope, topographic complexity, bioclimate, rainfall etc.......And an explanation on the choice of course.

Material and Methods. Study Area and Site selection What is meant by "Native vegetation" there can be native grasslands, native scrub, native bogs, native….. It does not mean native forest, the reader can be led to think that the "Native vegetation" on Figure 1, is the same as the Native forest on Figure 2, no true!

Material and Methods. Study Area and Site selection Why include here Figure 2? It looks like a result; in any case it should be moved and the plots clearly identified (meaning that the images on Figure 2 should correspond to 2 of the studied plots)

Material and Methods. Vegetation Survey The exotic forest plots were surveyed in the spring of 2023 within 20 x 20 meter plots, while the native forest plots were surveyed earlier, during the summer of 2012 and 2013, within larger 50 x 50 meter plots. This seems to be an insurmountable methodological problem with a very complex (impossible?) solution, a 10 year difference in the survey! Thats a decade, plants/trees do grow in 10 years. How can this be addressed when considering that other data such as "UAV canopy mapping" and "Terrestrial laser scanning" do not match the inventories? Even more taken into account that the authors state below that forests are homogeneous, were they 10 years ago? and then why should they still be homogeneous or even keep the same composition 10 years later?

Material and Methods. Vegetation Survey The exotic forest plots were surveyed in the spring of 2023 within 20 x 20 meter plots, while the native forest plots were surveyed earlier, during the summer of 2012 and 2013, within larger 50 x 50 meter plots. Why are the plots different in size?

Material and Methods. Vegetation Survey These surveys were designed following a standardised protocol (Borges et al. 2018). The protocol needs to be here described, but how can a protocol be applied in 2012 if it was published in 2018?

Material and Methods. Vegetation Survey The objective was to obtain comprehensive data on the composition of forest species. Eventually this statement should precede everything else.

Material and Methods. Vegetation Survey Due to the relative homogeneity of the native forests, no rarefaction process was applied to the data from these plots, allowing for the retention of all inventory data. This decision reflects the low variability in species composition within the native forest, ensuring a more accurate and robust comparison with exotic forest plots. This needs to be clearly explained, in any case it should belong to another part of the M & M, maybe data treatment? As it is corresponds to an empty sentence

Material and Methods. Vegetation Survey Species richness (H_0), representing the total number of species present in each plot, was recorded to evaluate biodiversity within the forest plots Therefore according to the authors species richness and biodiversity are the same concept? Does biodiversity increase with alien species? Is this a scientific statement or a political concession for managers of the Azorean landscape?

Material and Methods. Vegetation Survey Additionally, the basal area of trees with a diameter at breast height (DBH) greater than 10 cm was calculated in hectares. This variable provides an estimate of tree density and size, which is crucial for understanding forest biomass and its contribution to the overall structure of the forest. This sentence needs to be rewritten; the meaning is unclear.

Material and Methods. Vegetation Survey *Another important metric, species density, was quantified by counting the number of shoots per species and per square meter, allowing for an assessment of vegetation density in different areas. More information about the protocol can be found in (Borges et al. 2018).*Again at least partially the protocol should be detailed here, it was published in 2018 but the "native forest" plots surveyed 6 years before. This is confusing and not acceptable.

Material and Methods. Vegetation Survey *These measurements were further classified in three distinct forest strata: the ground layer, the understory, and the canopy, offering a vertical profile of the forest structure. Furthermore, species were categorized into four groups—endemic, native, introduced, and*

invasive—resulting in a total of twelve variables, with each stratum and species category providing specific insights into the forest's ecological composition and structure. I cannot understand this sentence, was DBH measured on the understory plants? How was density measured at the ground layer, just trees? Based on what did the authors segregate "introduced" and "invasive", a published list based on Azorean flora crossed with concepts like those published by Pyšek, Richardson and many others?

Material and Methods. Sites and lanscape characteristics *For each of the 18 study sites on Terceira Island, detailed data on elevation and landscape characteristics were collected to better understand the environmental factors influencing forest structure.* If the sites are not homogeneous in what concerns elevation and other geographical/ecological factors then how can they be compared?

Material and Methods. Sites and lanscape characteristics *Furthermore, landscape heterogeneity was quantified in the surrounding area of each site. Land use data, obtained from the Azorean government, were processed and analysed in QGIS. A quadrat of side 500 meters was designed around all plots, and the proportion of land use was assessed per category.* Data of the "Azorean government" should be of free access if so give an url on the text. This data are recent? Why not using the UAV to obtain the data? Is the classification of the "Azorean government" a clear match to the authors needs? See above the comment on Native vegetation and Native forest.

Material and Methods. Sites and landscape characteristics Table 1 might deserve a detailed comment, there are no invasive species on the ground layer

Material and Methods. Sites and landscape characteristics Table 1. Finally the reader understands that although there are references to arthropods and other types of non-plant diversity, this is a study of plant diversity, there are no other assessments being introduced on this manuscript. This a study of plants without reference to the actual plants, even less types of plant communities, very peculiar.

Material and Methods. Sites and landscape characteristics In fact what the authors seem to have studied are one peculiar type of native Forest (broad leaved although in the discussion *Juniperus brevifolia* is mentioned as dominant and this is not a broad leaf plant) and one type of exotic/invaded Forest (a Pittosporum dominated area, calling it forest another misleading concept)

Results *The characterisation of landscapes is a consequence of human accessibility and land-use*; this is what the authors decided to study not a conclusion of their study, in any case there no references to historical use of the Azorean forests on the introduction, a major issue since it clearly determines the framework of the study.

Results *The structural complexity of forest stands is derived from different sources in native and exotic woodlands*; what the authors are trying to say is that distinct "Natural" "successional" routes are being observed in distinct "forest", obviously they are different, they are historically distinct, the easily accessed areas had since the XV century many distinct uses, please read historical papers! They were initially cut for wood, them burned and cultivated for wheat then…… plants with Pittosporum and invaded by Pittosporum (and many other exotic plants), therefore the sources are human (potentially infinite, where are invading plants coming from, how many a year/decade?) or based on the few refugia (a concept not explored on the text). Further more what the authors refer as native (without description detail etc.) are mature forests? Secondary forests on permanent ecological/successional stands derived from there "complex orography", were they cut in the past (certainly they were although possibly not cultivated)

Results *The intrinsic complexity of native stands may be attributed to the traits of the few endemic tree species (e.g. the Azorean endemic cedar Juniperus brevifolia), which exhibit greater branch ramifications*, OR they can attributed to the fact that they or not the ecological equivalent of the Pittosporum stands, in order to solve this comundrum Native forests in areas not corresponding to

refugia should be included, it might even occur that the dominant species in smooth areas to be distinct species.

Results *The canopy of native forests shows greater structural and compositional homogeneity* If they represent simplified versions of the Native Forest due to historical use and refugia position this is exactly what we all expect to happen.

Statistical complexity does not solve the problems found in the original data, to the end of the manuscript the biological descriptors are unclear, vascular plants? Vascular and Bryophytes? Arthropods?
