# Supplementary material for: Landscape to microhabitat: Uncovering the multiscale complexity of native and exotic forests on Terceira Island (Azores, Portugal)
Source: PLoS One. 2025 Jun 16;20(6):e0326304. doi: 10.1371/journal.pone.0326304 (PMC12169593; doi:10.1371/journal.pone.0326304)
Supplement: Attachment — Submitted filename: Reviews comments V1_resubmission.docx [file pone.0326304.s004.docx]

# Journal Requirements:

When submitting your revision, we need you to address these additional requirements.

1. Please ensure that your manuscript meets PLOS ONE's style requirements, including those for file naming. The PLOS ONE style templates can be found at

<https://journals.plos.org/plosone/s/file?id=wjVg/PLOSOne_formatting_sample_main_body.pdf>

and

<https://journals.plos.org/plosone/s/file?id=ba62/PLOSOne_formatting_sample_title_authors_affiliations.pdf>

We have formatted the manuscript according to the guidelines provided in the PLOS ONE style templates, including the file naming conventions.

2.  In your Methods section, please provide additional information regarding the permits you obtained for the work. Please ensure you have included the full name of the authority that approved the field site access and, if no permits were required, a brief statement explaining why.

We have added the requested information regarding the permits in the Methods section.

3. Thank you for stating the following in the Acknowledgments Section of your manuscript:
“S.L. is funded by the Azorean Government Ph.D. grant numbers M3.1.a/F/012/2022. R.B.E, RG and P.A.V.B. are currently funded by the projects Azores DRCT Pluriannual Funding (M1.1.A/INFRAEST CIENT/001/2022) and FCT—Fundação para a Ciência e Tecnologia FCT[1]UIDB/00329/2020-2024 DOI 10.54499/UIDB/00329/2020 (Thematic Line 1—integrated ecological assessment of environmental change on biodiversity). D.S received no outside funding.”

We note that you have provided funding information that is not currently declared in your Funding Statement. However, funding information should not appear in the Acknowledgments section or other areas of your manuscript. We will only publish funding information present in the Funding Statement section of the online submission form.
Please remove any funding-related text from the manuscript and let us know how you would like to update your Funding Statement. Currently, your Funding Statement reads as follows:
“S.L. is funded by the Azorean Government Ph.D. grant numbers M3.1.a/F/012/2022.

R.B.E, RG and P.A.V.B. are currently funded by the projects Azores DRCT Pluriannual Funding (M1.1.A/INFRAEST CIENT/001/2022) and FCT—Fundação para a Ciência e Tecnologia FCT-UIDB/00329/2020-2024 DOI 10.54499/UIDB/00329/2020 (Thematic Line 1—integrated ecological assessment of environmental change on biodiversity).

D.S received no outside funding.”

Please include your amended statements within your cover letter; we will change the online submission form on your behalf.

We corrected the manuscript accordingly. The Funding Statement you provided is correct.

4. Thank you for stating the following financial disclosure:
“S.L. is funded by the Azorean Government Ph.D. grant numbers M3.1.a/F/012/2022.

R.B.E, RG and P.A.V.B. are currently funded by the projects Azores DRCT Pluriannual Funding (M1.1.A/INFRAEST CIENT/001/2022) and FCT—Fundação para a Ciência e Tecnologia FCT-UIDB/00329/2020-2024 DOI 10.54499/UIDB/00329/2020 (Thematic Line 1—integrated ecological assessment of environmental change on biodiversity).

D.S received no outside funding. “

Please state what role the funders took in the study. If the funders had no role, please state: "The funders had no role in study design, data collection and analysis, decision to publish, or preparation of the manuscript."
If this statement is not correct you must amend it as needed.
Please include this amended Role of Funder statement in your cover letter; we will change the online submission form on your behalf.

We added the statement that our funders did not have any role in the Funding Statement.

5. In the online submission form, you indicated that “Data are available on request.”

All PLOS journals now require all data underlying the findings described in their manuscript to be freely available to other researchers, either 1. In a public repository, 2. Within the manuscript itself, or 3. Uploaded as supplementary information.
This policy applies to all data except where public deposition would breach compliance with the protocol approved by your research ethics board. If your data cannot be made publicly available for ethical or legal reasons (e.g., public availability would compromise patient privacy), please explain your reasons on resubmission and your exemption request will be escalated for approval.

We have addressed the data availability requirements by adding all data used in the manuscript as supplementary materials.

# Review Comments to the Author

## Reviewer #1:

 The manuscript PONE-D-24-59937 (“Landscape to Microhabitat: Uncovering the Multiscale Complexity of Azorean Forests”) identify the structural and compositional differences between native and exotic woodlands on Terceira Island, Azores.

The manuscript is correct and it is good. The references is very good and the history very well explained and its justification. The work is very interesting.

The introduction and methodology is correct but it’s necessary some annotations (see below). With respect to the results, I see it as good, although is necessary basic information. Finally, in the discussion section, it was very well written.

Some annotations:
With respect to material and methods, the plots area (20 x 20 m) are enough? What determines the size of the plot? The maximum height of the trees?

Thank you for your positive feedback on our manuscript. Regarding the plot size in the Materials and Methods section, the 20 x 20 m plots were determined based on accessibility and logistical considerations. Due to the small stature of Azorean forests, plots sizes usually range from 5x5 m to 15 x 15 m (e.g. Dias et al. 2011; Elias et al. 2011; Elias et al. 2016. Additionally, the empirical assessment of the homogeneity of the surroundings at the center of the plot justified the selection of this plot size.

The authors could considered include table 1 as supplementary material.

Figure 3 needs improvement.

Thank you for your suggestions. We have moved Table 1 to the supplementary material and added this mention in the beginning of the paragraph. Additionally, we have improved the visual quality of Figure 3 and incorporated some critical information from the former Table 1 into the figure for better clarity. Please note that figure 3 become figure 2 as the former figure 2 was moved to the results based on the comment of the second referee.

It would be interesting for the authors to include a list of the species present in each plot and in each treatment (native and exotic woodlands) as supplementary material indicating, if possible, their origin species (native, endemic, introduced, invasive).

Following the editor's guidelines, we have now provided all the data used in the analysis as supplementary materials. This includes a list of the species present in each plot and treatment (native and exotic woodlands), along with their colonization origin based on the updated information available in AZORESBIOPORTAL (native non-endemic, endemic, introduced, invasive).

In addition, it would be good to include a short introduction to the results with a paragraph with the percentages of species present in each type of forest.

We have added brief descriptive sentences at the beginning of the Results section as well as a table summarising our surveys.

In the discussion, the authors indicates that were ten plot in native woodlands but really are nine (Second paragraph in “The characterization of landscapes is a consequence of human accessibility and land-use”)

Thank you for pointing out the discrepancy. We have corrected the typo in the discussion section. Initially, ten plots were selected, but one plot was removed from the study due to the inability to obtain LiDAR data for it, resulting in a total of nine plots in the native woodlands.

## Reviewer #2:

Abstract … By analysing landscape, habitat, and microhabitat traits, we found that native forests are situated in less accessible terrains…. rephrase, the location of native forests is not a trait, or derived from the fact that they are native, just the opposite, they are the remains of a former native forest covering all the locations.

We have revised the abstract to clarify that the current locations of native forests in less accessible terrains are not inherent traits of these forests.

Abstract … homogeneous structural complexity derived from endemic vascular plant species…. I do not agree with "derived", it should be "and", adding enough exotic species or letting succession into the non-native forest will eventually result in complexity, complexity does not derive from endemicity (see comments on the Results).

What we meant was that endemic trees, such as the dominant Juniperus brevifolia, exhibit a branching architecture that directly contributes to the structural complexity observed in native forests. To clarify this point, we have revised the text accordingly.

Abstract ...exotic forests, which are situated in human-modified landscapes; Exotic woodlands are the human modified landscape, rephrase as "exotic forests, are the result of human actions, afforestation or introduction of invading tree plants.

Thank you for your suggestion. The plots are in sites where there was afforestation followed by agricultural use that was latter abandoned, allowing the invasion of exotic tree species. We have rephrased the sentence to clarify that exotic forests result from human actions, including afforestation or the introduction of invasive tree species.

Abstract ...The ground and canopy layers in exotic forests were more invaded by nonindigenous species, while the understory demonstrated greater resilience... very confusing statement, first an absolute need to resolve the difference between afforested areas and invaded areas, then also to differential use and actions of both, usually afforested areas have periodical cleaning of the understory?

We acknowledge the importance of distinguishing between afforested and invaded areas. Based on our knowledge, nearly all exotic forest plots in this study were originally afforested for orchards or vineyards and later abandoned. Currently, there is no evidence of ongoing human intervention in these areas or their surroundings. However, we recognize the limitation that precise historical land-use data are not available. We also have clarified the point in the manuscript that the understory layer considered in this study is richer in terms of native species comparing to the ground and canopy layers.

Abstract ...Our findings suggest a temporal progression of forest change, from pristine native to disturbed exotic stands.; The authors have no follow up of the dynamics of the forest to support this statement, by contrary many references exist proving the Azores forest cover suffered a massive and historical change, the object of study are the results of the change, conclusions cannot be to prove the chosen model of exotic versus native exists, something that should be stated in the abstract as historical, but by contrary to explain the complexity, diversity etc of the 2 distinct “woodlands”

We agree and have rephrased the statement to remain more factual, emphasizing the historical context of forest change in the Azores and focusing on the structural and ecological characteristics of the two distinct woodland types.

Abstract “...These insights highlight the necessity for scale-specific conservation, prioritising native forests in remote areas and controlling invasive species in exotic forests to enhance ecological resilience”; basically what the authors propose is to protect what already is protected not because is legally protected but because is as stated "in less accessible terrains" and to keep the exotic areas as exotic although this exotic are themselves invasive, this is an absurd

We acknowledge the need to clarify our statement and have rephrased it to emphasize the importance of concrete conservation actions and continuous monitoring. While protected areas may be designated, their effective management and enforcement are essential to preserving native forests and controlling invasive species in exotic woodlands. In fact, we still see many areas in the archipelago (eg. In Flores, São Jorge and Faial) where the protection is only in the paper. Cattle is still moving around freely. This revision ensures a more action-oriented perspective in our conclusions.

Introduction The structure of the introduction is inverted, the object of study should firstly be defined and then the first 3 paragraphs on the first page should follow.

Thank you for your suggestion. However, we have chosen to maintain the current structure of the introduction, as it follows a standard scientific writing approach—first presenting the broader context of the study before defining its specific objectives. We believe this approach enhances the clarity of the narrative, providing a coherent and logical progression of ideas that guides the reader from general background information to the precise aims of the research.

Introduction The Azores, one of the most remote archipelagos in the North Atlantic Ocean, is made up of nine islands where forests contain a mix of native and exotic species, shaped by both natural processes and six centuries of human influence. (Elias et al. 2016). This or a modified version of this sentence should be the opening sentence of the Introduction, the phrase is however overly optimistic in the use of the concept of "Mosaic" or “Mix”, the Azores have certainly less than 5% of Native forest cover and therefore a Mosaic of 5% versus 95% is not a mosaic. This needs to be clearly expressed by the authors on the introduction. Several studies published by one of the authors (Elias) can be used to state and support this key point to the readers.

We have rephrased "mosaic" to "patches" to more accurately reflect the limited extent of native forest cover in the Azores. Additionally, we have clarified this point in the introduction, citing relevant studies, to ensure readers understand the significant imbalance between native and exotic forest areas.

Introduction ...consist primarily of broad-leaved evergreen trees. There are according to Elias (2016), Dias et al. (2021), and others several types of vegetation including some that are not broad-lead. To characterize the broad-leaved evergreen trees as such without any further hint or name is insufficient; include also the references: Dias, Eduardo; Mendes, Cândida; Aguiar, Carlos (2021). Vegetação dos Açores. In Capelo, J.; Aguiar, C. (Eds.), Vegetação de Portugal. Lisboa: INCM, p. 155-179. ISBN 978-972-27-2879-9; Mucina, L.; Bültmann, H.; Dierßen, K.; Theurillat, J.-P.; Raus, T.; Carni, A.; Šumberová, K.; et al. "Vegetation of Europe: hierarchical floristic classification system of vascular plant, bryophyte, lichen, and algal communities". Applied Vegetation Science 19 (2016): 3-264; Fernández Prieto, J.C.; Aguiar, C.; Dias, E.; Fernández Prieto, J.A.; Aguiar, Carlos; Dias, Eduardo; FernÃ¡ndez Prieto, JosÃ© Antonio. "Description of some new syntaxa from the Azores archipelago". International Journal of Geobotanical Research 2 1 (2012): 111-116. <http://dx.doi.org/10.5616/ijgr120007>

We agree that a more detailed characterization of the broad-leaved evergreen trees in the Azores is important. We have included peer-reviewed relevant references to clarify the diversity of native vegetation types in the archipelago. Additionally, we have provided a clear definition of the native woodlands considered in this study, which aims to provide a broad overview of the structural complexity of the main woodlands and their potential determinants.

Introduction ...many endemic taxa Characterize "Many", or compare with other similar forests, however we lack the sense/definition of forest and therefore a way to compare with other similar forests. There is reference in this introduction characterizing the types of forest and giving proper biogeographical framework.

We cited several references that detail the endemic species composition, including recent and updated checklists. To address your comment, we have now included a clear definition of the native vegetation type considered in this study in the Materials and Methods section, which will help provide a better framework for comparing with similar forests.

Introduction ...As a result, the Azores’s landscape is now a mosaic of native and exotic forests, each playing a different ecological role… This is a bold statement and a conclusion, furthermore without references, see above the comment on the "Mosaic", do invading exotic plants play a role is this a political or a scientific statement?

We have rephrased "role" to "importance" to make the statement more precise. Additionally, we acknowledge that this sentence is broad, but it is further detailed in the following sentences, which include relevant references. We have also addressed your earlier comment regarding the term "mosaic."

Introduction ...Native forests are crucial for maintaining the Azorean island’s biodiversity, acting as refuges for endemic plants and animals (Lhoumeau and Borges 2023) Well… native forests are native in fact.

Thank you for your comment. You are correct that native forests are, by definition, native. We included this distinction to differentiate them from exotic woodlands resulting from human activity. Additionally, we have rephrased "refuges" to "home" to reflect the pristine nature of native forests more accurately.

Introduction Finally, the vegetation data collected from surveys provides information on species composition and density across all layers. The data, gathered on Terceira Island, are used to address the following question: What are the defining characteristics of the vegetation structure of the various types of Azorean forests, with a particular focus on the native and exotic woodlands? By answering this question, this study aims to provide a detailed characterisation of Terceira's forests, offering insights into how both native and exotic forest types are integrated within the island's broader ecosystem. The reader finds out that after all the study concerns one island out of nine islands, that needs to be addressed on the proposed title "Landscape to Microhabitat: Uncovering the Multiscale Complexity of Terceira Island forests (Azores, Portugal)" but see further comments below.

We agree that the study focuses on Terceira Island, and we have clarified this point in the introduction by highlighting that Terceira is the island with the best-preserved and most representative native vegetation of the Azores archipelago, supported by relevant references. However, we have decided to retain the original title, as it reflects the broader multiscale approach of the study, which is still applicable to Terceira's forests in this context.

Material and Methods. Study Area and Site selection The second part of the first paragraph needs to be moved to the introduction (to a new and so far lacking characterization of the Terceira forests and habitats)

Material and Methods. Study Area and Site selection The sentence "the third largest of the nine islands in the archipelago (figure 1)." should be moved to the first paragraph

Thank you for the suggestion. The relevant portion of the paragraph has been moved to the introduction and smoothly integrated to provide a more detailed characterization of Terceira's forests and habitats.

Material and Methods. Study Area and Site selection The sentence "which are characterised by broadleaved evergreen trees and are home to a variety of endemic plant species" is conclusive and derives from the results themselves should deleted.

The sentence has been revised and replaced with a description of the native vegetation type considered in this study providing a clear delimitation of the meaning of native woodlands considered in the manuscript.

Material and Methods. Study Area and Site selection What is meant by "by exotic vegetation, primarily consisting of Pittosporum undulatum", Pittosporum undulatum is an invasive plant species very well known, documented etc. therefore 1) there should be detailed information on this species on the introduction, including reference to many publications concerning the Azores/Pittosporum undulatum question; 2) Pittosporum undulatum is an Invasive tree species and should be delt as invasive; 3) if there are no other dominant exotic species referred on the study (such as Cryptomeria japonica, the dominant exotic tree in the Azores... then the title of the manuscript should be further adapted/changed to fit this much more simple comparison. One Island and Native (even this can be explicit) versus Pittosporum invaded areas.

We have included detailed information about Pittosporum undulatum in the discussion section, where it is used to interpret the results, rather than in the context of the study. We believe this placement provides a clearer focus on the study's objectives.

We have treated Pittosporum undulatum as an invasive species in our species survey, as it is well-documented as such in the Azores.

Cryptomeria is not the dominate exotic species. Pittosporum dominated areas occupy 33% of forest surface in the Azores. Production forests (mostly, but not only (!), of Cryptomeria) occupy 31%. This is according to the «State of the Environment Report of 2023» (Azorean Regional Government). Therefore, while Pittosporum undulatum is the dominant exotic species in our dataset, we have chosen to retain the original title. This is because Pittosporum dominates most exotic woodlands across the Azores. Cryptomeria, on the other hand, typically appears in specific production areas, which are distinguished in the literature (see Borges Silva LC, Pavão DC, Elias RB, Moura M, Ventura MA, Silva L (2022) Taxonomic, structural diversity and carbon stocks in a gradient of island forests. Scientific Reports 12: 1038. <https://doi.org/10.1038/s41598-022-05045-w> ). Therefore, we believe the title still accurately reflects the broader focus of our study.

Material and Methods. Study Area and Site selection There is a lacking explanation on the 18 plots choice, it cannot just be because they are already in use in something else. Are they bioclimaticaly homogeneous? Are they similar in use, in historical use? Etc. A table with all the plots and as many characteristics as possible should be included, namely altitude, slope, topographic complexity, bioclimate, rainfall etc.......And an explanation on the choice of course.

We have provided a comprehensive compilation of the available data regarding the plots in the supplementary material. The choice of sites was primarily based on their use in numerous previous studies, as they are established as permanent plots for long-term monitoring (see Borges et al., 2018; GIMs Protocol), which allowed us to minimize further disturbance to areas of native vegetation. Additionally, all sites within each forest type are homogeneous in terms of vegetation type, as previously described.

Material and Methods. Study Area and Site selection What is meant by "Native vegetation" there can be native grasslands, native scrub, native bogs, native….. It does not mean native forest, the reader can be led to think that the "Native vegetation" on Figure 1, is the same as the Native forest on Figure 2, no true!

We have clarified the terms "native vegetation" and "native forest" throughout the manuscript to ensure that the distinction is clear to the reader. This clarification is also reflected in the figure captions to avoid any confusion between the two terms.

Material and Methods. Study Area and Site selection Why include here Figure 2? It looks like a result; in any case it should be moved and the plots clearly identified (meaning that the images on Figure 2 should correspond to 2 of the studied plots)

Thank you for your suggestion. The figure has been moved to the results section and smoothly integrated there. Additionally, information identifying the plots has been added to ensure clarity, and the images in Figure 2 now correspond to two of the studied plots.

Material and Methods. Vegetation Survey The exotic forest plots were surveyed in the spring of 2023 within 20 x 20 meter plots, while the native forest plots were surveyed earlier, during the summer of 2012 and 2013, within larger 50 x 50 meter plots. This seems to be an insurmountable methodological problem with a very complex (impossible?) solution, a 10 year difference in the survey! Thats a decade, plants/trees do grow in 10 years. How can this be addressed when considering that other data such as "UAV canopy mapping" and "Terrestrial laser scanning" do not match the inventories? Even more taken into account that the authors state below that forests are homogeneous, were they 10 years ago? and then why should they still be homogeneous or even keep the same composition 10 years later?

We chose to use the native forest surveys from 10 years ago for several reasons:

The data were collected as part of the NETBIOME - ISLANDBIODIV project, providing valuable long-term information.

We did not observe any significant changes in the overall structure of the woodlands, as they are mature forests under protection. In fact, within the scope of the MOVECLIM Project other permanent plots are been resurveyed by some of the authors of these manuscript. Some of these plots are in Juniperus-Ilex forests of Terceira. They were resurveyed in 2024, and no significant structural changes were observed.

Conducting new surveys could risk altering the forest structure due to trampling, which is particularly important since the focus of this study is on the structural complexity of the forests.

Similarly, while the inventory and remote sensing surveys do not match temporally, we did not observe any dramatic changes in forest structure. The primary aim of our study is to provide a broad, comparative overview of the structure of both native and exotic woodlands, and we believe the 10-year difference does not undermine this objective.

Material and Methods. Vegetation Survey The exotic forest plots were surveyed in the spring of 2023 within 20 x 20 meter plots, while the native forest plots were surveyed earlier, during the summer of 2012 and 2013, within larger 50 x 50 meter plots. Why are the plots different in size?

The larger 50 x 50 meter plots for the native forests were chosen during the design of the ISLANDBIODIV project and we use the data gathered. The 20 x 20 meter plots in the exotic forests were selected based on forest height, accessibility and logistical considerations. Furthermore, the homogeneity of the plots supported the selection of this area to represent the forest structure being studied.

Material and Methods. Vegetation Survey These surveys were designed following a standardised protocol (Borges et al. 2018). The protocol needs to be here described, but how can a protocol be applied in 2012 if it was published in 2018?

Thank you for pointing this out. The vegetation survey protocol was initially designed during earlier projects, notably ISLANDBIODIV and MOVECLIM, and was implemented in 2012. The formal publication of the protocol in 2018 was part of a broader effort, which included contributions from multiple specialists and research groups, leading to a delay in its official release.

Material and Methods. Vegetation Survey The objective was to obtain comprehensive data on the composition of forest species. Eventually this statement should precede everything else.

Thank you for your suggestion. We agree and have moved the sentence to the beginning of the section

Material and Methods. Vegetation Survey Due to the relative homogeneity of the native forests, no rarefaction process was applied to the data from these plots, allowing for the retention of all inventory data. This decision reflects the low variability in species composition within the native forest, ensuring a more accurate and robust comparison with exotic forest plots. This needs to be clearly explained, in any case it should belong to another part of the M & M, maybe data treatment? As it is corresponds to an empty sentence

We have removed the paragraph to ensure consistency and clarity.

Material and Methods. Vegetation Survey Species richness (H₀), representing the total number of species present in each plot, was recorded to evaluate biodiversity within the forest plots Therefore according to the authors species richness and biodiversity are the same concept? Does biodiversity increase with alien species? Is this a scientific statement or a political concession for managers of the Azorean landscape?

We have rephrased the paragraph to clarify that species richness (H₀) refers specifically to the total number of species in each plot as it is defined as a measure of diversity in Hill MO (1973) Diversity and Evenness: A Unifying Notation and Its Consequences. Ecology 54: 427–432. <https://doi.org/10.2307/1934352>.

Material and Methods. Vegetation Survey Additionally, the basal area of trees with a diameter at breast height (DBH) greater than 10 cm was calculated in hectares. This variable provides an estimate of tree density and size, which is crucial for understanding forest biomass and its contribution to the overall structure of the forest. This sentence needs to be rewritten; the meaning is unclear.

We have rephrased the sentence for clarity

Material and Methods. Vegetation Survey Another important metric, species density, was quantified by counting the number of shoots per species and per square meter, allowing for an assessment of vegetation density in different areas. More information about the protocol can be found in (Borges et al. 2018). Again at least partially the protocol should be detailed here, it was published in 2018 but the "native forest" plots surveyed 6 years before. This is confusing and not acceptable.

We have added more details about the protocol to clarify its application. Although the protocol was formally published in 2018, it was developed and implemented in earlier surveys, including those for the native forest plots, as part of prior projects

Material and Methods. Vegetation Survey These measurements were further classified in three distinct forest strata: the ground layer, the understory, and the canopy, offering a vertical profile of the forest structure. Furthermore, species were categorized into four groups—endemic, native, introduced, and invasive—resulting in a total of twelve variables, with each stratum and species category providing specific insights into the forest’s ecological composition and structure. I cannot understand this sentence, was DBH measured on the understory plants? How was density measured at the ground layer, just trees? Based on what did the authors segregate "introduced" and "invasive", a published list based on Azorean flora crossed with concepts like those published by Pyšek, Richardson and many others?

We have provided clearer details on the protocol and the classification of species. The classification of species into "endemic," "native," "introduced," and "invasive" is based on Borges PAV, Costa A, Regina C, Gabriel R, Gonçalves V, Frias Martins A, Melo I, Parente M, Raposeiro P, Rodrigues P, Serrão Santos R, Silva L, Vieira P, Vieira V (2010) A list of the terrestrial and marine biota from the Azores. Principia, Parede. It compiles data from various sources and more recent updates available in AZORES BIOPORTAL

Regarding your questions, DBH was only measured for tree species. Density measurements for the ground layer were based on the number of shoots of woody species per square meter. We have clarified these points to ensure the methodology is more transparent.

Material and Methods. Sites and lanscape characteristics For each of the 18 study sites on Terceira Island, detailed data on elevation and landscape characteristics were collected to better understand the environmental factors influencing forest structure. If the sites are not homogeneous in what concerns elevation and other geographical/ecological factors then how can they be compared?

We have removed part of the sentence. While the sites vary in terms of elevation and other geographical/ecological factors, this variability does not pose a methodological issue. Instead, it offers valuable information about the distribution of these forest stands, which helps to better characterize their structural diversity.

It is important to clarify that the goal of our study is to provide a general overview of the forest structure.

Material and Methods. Sites and lanscape characteristics Furthermore, landscape heterogeneity was quantified in the surrounding area of each site. Land use data, obtained from the Azorean government, were processed and analysed in QGIS. A quadrat of side 500 meters was designed around all plots, and the proportion of land use was assessed per category. Data of the "Azorean government" should be of free access if so give an url on the text. This data are recent? Why not using the UAV to obtain the data? Is the classification of the "Azorean government" a clear match to the authors needs? See above the comment on Native vegetation and Native forest.

We have added the data source for the land use information, which is publicly available through the Azorean government. This dataset was used due to its accessibility and suitability for our needs, which is why we did not use UAV data for this purpose. While the classification of the Azorean government data may not align perfectly with our definitions of "native vegetation" and "native forest," it provided a reliable and practical basis for assessing landscape heterogeneity around the study sites.

Material and Methods. Sites and landscape characteristics Table 1 might deserve a detailed comment, there are no invasive species on the ground layer

In response to other reviewer feedback, Table 1 has been moved to the supplementary material. Additionally, we have included a new table summarizing species composition at the beginning of the results section to provide a clearer overview of the vegetation structure, including the presence of invasive species across different forest strata.

Material and Methods. Sites and landscape characteristics Table 1. Finally the reader understands that although there are references to arthropods and other types of non-plant diversity, this is a study of plant diversity, there are no other assessments being introduced on this manuscript. This a study of plants without reference to the actual plants, even less types of plant communities, very peculiar.

As clearly stated in the study's objectives, our focus is on assessing differences in structural complexity between native and exotic woodlands. While plant diversity is an inherent part of forest structure, our primary aim is to characterize and compare the physical and compositional attributes of these forest types rather than conduct a taxonomic or community-level study.

Material and Methods. Sites and landscape characteristics In fact what the authors seem to have studied are one peculiar type of native Forest (broad leaved although in the discussion Juniperus brevifolia is mentioned as dominant and this is not a broad leaf plant) and one type of exotic/invaded Forest (a Pittosporum dominated area, calling it forest another misleading concept)

Throughout the manuscript, we have clarified the definitions of our study objects to avoid any ambiguity. Our goal is to provide a broad and general overview of the main and most representative woodlands on Terceira Island. Juniperus brevifolia was a key component of native forests and we added this information. Similarly, we acknowledge that Pittosporum undulatum forms dense invaded stands, which we refer to as exotic woodlands for consistency in our comparisons.

Results The characterisation of landscapes is a consequence of human accessibility and land-use; this is what the authors decided to study not a conclusion of their study, in any case there no references to historical use of the Azorean forests on the introduction, a major issue since it clearly determines the framework of the study.

Historical land-use data for the Azorean forests are not precisely documented. However, we address the past use of the land, particularly in relation to exotic woodlands, within the discussion section. To better reflect the scope of this analysis, we have also rephrased the title of the paragraph accordingly.

Results The structural complexity of forest stands is derived from different sources in native and exotic woodlands; what the authors are trying to say is that distinct “Natural” “successional” routes are being observed in distinct “forest”, obviously they are different, they are historically distinct, the easily accessed areas had since the XV century many distinct uses, please read historical papers! They were initially cut for wood, them burned and cultivated for wheat then…… plants with Pittosporum and invaded by Pittosporum (and many other exotic plants), therefore the sources are human (potentially infinite, where are invading plants coming from, how many a year/decade?) or based on the few refugia (a concept not explored on the text). Further more what the authors refer as native (without description detail etc.) are mature forests? Secondary forests on permanent ecological/successional stands derived from there “complex orography”, were they cut in the past (certainly they were although possibly not cultivated)

We have clarified the definition of the studied woodlands throughout the manuscript, addressing your previous comment. We acknowledge that the structural complexity of native and exotic woodlands results from distinct historical and ecological trajectories, including human influences and natural succession. Additionally, we recognize the role of refugia and have refined the descriptions of the forest types to better reflect their ecological and historical backgrounds.

Results The intrinsic complexity of native stands may be attributed to the traits of the few endemic tree species (e.g. the Azorean endemic cedar Juniperus brevifolia), which exhibit greater branch ramifications, OR they can attributed to the fact that they or not the ecological equivalent of the Pittosporum stands, in order to solve this comundrum Native forests in areas not corresponding to refugia should be included, it might even occur that the dominant species in smooth areas to be distinct species.

We acknowledge the concern about the ecological equivalence of the studied woodlands. However, our study aims to provide a broad comparative overview of the structural complexity between native and exotic woodlands rather than a precise one-to-one comparison. While refugia may influence native forest structure, our focus remains on characterizing the overall patterns of complexity in these dominant forest types on Terceira Island. Moreover, in Terceira there no such areas available anymore.

Results The canopy of native forests shows greater structural and compositional homogeneity If they represent simplified versions of the Native Forest due to historical use and refugia position this is exactly what we all expect to happen.
Statistical complexity does not solve the problems found in the original data, to the end of the manuscript the biological descriptors are unclear, vascular plants? Vascular and Bryophytes? Arthropods?

Our study is centered on assessing the structural complexity of native and exotic woodlands, with a clear focus on vascular plant composition. Our primary objective is to characterize the overall structural differences rather than reconstruct past ecological processes. We have clarified our data scope and biological descriptors in the manuscript to ensure transparency in our methodological approach.

## Reviewer #3:

Dear authors

After throughout analysis of the manuscript (MS), my general opinion is that the research question is interesting, but, despite analytical workflow being adequate, there are some flaws that may compromise the coherence of the MS. Also, the results are somewhat self-evident, for instance that natural/pristine forest is structurally more complex than artificial/exotic forest stands. Besides I would expect that some possible analysis of functional diversity between the two types of forest as the result of different history and drivers could have been addressed by author.

Your feedback is greatly appreciated. While it is true that the structural differences between native and exotic forests might seem self-evident, our study is the first to quantitatively assess objectively these differences using LiDAR technology in the Azores archipelago. This novel approach allows for a high-resolution analysis of forest structure, providing empirical evidence that was previously lacking in the literature. By applying standardized remote sensing techniques, we ensure that the structural complexity of these forest types is rigorously compared.

We acknowledge that functional diversity plays a key role in shaping forest structure, as species traits such as branching patterns, leaf morphology, and growth form directly influence layers complexity and stratification. While we briefly discuss these aspects, a comprehensive functional diversity analysis requires additional species-specific trait data and a distinct analytical framework. Given our focus on broad-scale structural comparisons, such an analysis falls beyond the scope of this study but represents an important avenue for future research. Our results lay the groundwork for more detailed investigations into the functional implications of forest structure, potentially guiding future conservation and management efforts in the Azores.

In concrete, I may point some of the flaws or aspects that are unclear I think should be addressed in a futures version of the MS.
1. The sample is too small to be representative. I understand that logistic effort might restrain the dimension of the sample buu, 18 plots hardly represent the ecological variability of the Azorean Forest.

Your concern regarding sample size is valid, and we acknowledge that 18 plots cannot fully capture the ecological variability of all Azorean forest types. However, our study aims to provide a general comparative assessment of forest structural complexity rather than an exhaustive characterization of all forest types in the region. While additional plots could further strengthen our conclusions, our findings still offer valuable insights into the contrasting structural properties of native and exotic woodlands in the Azores, highlighting key patterns that warrant further investigation. Our approach will also be an inspiration for other island studies, offering a framework to explore forest structural complexities and ecological patterns in different island ecosystems, ultimately contributing to a broader understanding of biodiversity dynamics in isolated environments.

To address this limitation, we have refined our definition of the studied forest types, particularly regarding native vegetation, ensuring that our comparisons are ecologically meaningful. Also, within each forest type—such as the Juniperus-Ilex forests—we empirically observed a relatively high degree of homogeneity, which was later supported by our structural data analyses.

2. It is very unclear the exact dates of sampling, because authors state that sampling has started in 2012’ and could mean that 13 plots were sampled at that time or thenceforth (when exactly?). Some sampling was made already in the summer of 2024. Therefore, part of the (already small) sample was made with an interval of maybe 12 years. This means that ecological succession may have led to composition and structure changes and some soil use change could have happened. I think you cannot compare plots with such a time span without accounting for time, which authors did not in their analysis.

We acknowledge the concerns regarding the temporal differences in data collection. To clarify, vegetation survey in the native forest plots were done the summer of 2013, while the exotic forest plots were surveyed in the spring of 2024. For both, LiDAR and UAV data collection were performed in summer 2024.

We recognize that ecological succession can alter forest composition and structure over time. However, our choice to use the2013 data for native forests was driven by the availability of high-quality vegetation surveys conducted as part of previous research projects (e.g., ISLANDBIODIV project). Additionally, these native forests are mature and protected, with minimal anthropogenic disturbances, and empirical observations suggest that their overall structure and composition has remained stable over the past decade.

While temporal differences are a limitation, our primary objective is to provide a broad comparison of forest structure between native and exotic woodlands. To mitigate potential biases, we focus on structural attributes rather than fine-scale compositional dynamics. However, we acknowledge that incorporating a temporal dimension in future studies would provide a more comprehensive understanding of long-term forest dynamics.

Another unclear aspect is the discrepancy of dates of the survey of metrics: the method reference (Borges, 2018) is posterior to the putative dates of the survey (2012, 2013). I really have a difficulty with understanding what procedure was really used at the time.

The vegetation survey protocol used in 2013 was originally developed as part of earlier research projects, such as ISLANDBIODIV . The methodology was standardized and widely applied in multiple studies before being formally published in Borges et al. (2018).

The 2018 publication represents a consolidation and refinement of protocols that had already been in use. The core methodology used in our study remained consistent with these earlier protocols, ensuring comparability between datasets. However, we acknowledge the need for greater clarity on this point and have revised the manuscript.

3. The detailed technical description of devices and procedure of canopy survey by LIDAR is superfluous and cumbersome in the main text and should be either omitted or sent to supplementary materials.

We maintain that the detailed description of LiDAR data collection and processing is essential to the manuscript, as it constitutes a fundamental component of our study. Given the increasing use of LiDAR in vegetation structure analysis, we believe that many researchers will benefit from a clear and transparent methodology. Providing detailed technical descriptions allows for reproducibility and ensures that our approach can be critically evaluated and replicated by others in similar studies.

4. I would like to see in a table how the parameters sampled in the surveys were converted to concrete trait variables and their scale values. The table presenting the 49 variables dos does not do this.

To improve clarity and accessibility, we have moved the original Table 1 to the supplementary material. Additionally, we have enhanced Figure 3 (now Figure 2) to better illustrate the grouping of variables and their ecological significance in our study. This revision ensures that readers can more easily understand how the surveyed parameters were translated into concrete trait variables and their respective scales.

5. The sampling of plant species simply classified into ‘native’ and ‘exotic/invasive’ is a very poor floristic classification of plant species. I think it is hard to understand and overly simplistic.

We acknowledge that a simple classification of plant species into "native" and "exotic/invasive" may oversimplify the ecological complexity of these forests. The classification follows the data from a published checklist of Azorean biota (see Borges PAV, Costa A, Regina C, Gabriel R, Gonçalves V, Frias Martins A, Melo I, Parente M, Raposeiro P, Rodrigues P, Serrão Santos R, Silva L, Vieira P, Vieira V (2010) A list of the terrestrial and marine biota from the Azores. Principia, Parede.). This checklist compiles multiple sources and provides a standardized categorization of species.

6. The use of arbitrary surface for plots for the sake of homogeneity, not using minimal-area/rarefaction procedure is unjustified. Rarefaction can be used once for each forest type then the surface value used repeatedly.

This part of the data analysis was removed to enhance clarity. Our primary concern was the difference in plot size between vegetation surveys, which could have influenced vascular plant community analysis. However, as previously acknowledged, the observed homogeneity within each forest type mitigates the issue of plot size differences, making rarefaction unnecessary for our study objectives.

7. The idea of indirect ordination by NMDS and then analysing driver correlates by Spearman correlation with axes is a good idea, although there are direct methods (canonical correlation or canonical correspondence analysis) that might have bee used instead. The real problem – I have serious difficulties in understanding – is that authors seem to have performed the analysis on a matrix with all the variables (!?). And then candidate explanatory variables were re-used (with Spearman correlation) to interpret NMDS axis? The procedure is not clear in the MS.

The paragraph was moved in the manuscript. For each site, we correlate the 2D positions in the global NMDS space (current figure 3) with the corresponding 49D positions from the original dataset. This allows us to assess the relationship between the ordination axes and the candidate explanatory variables. We have improved the clarity of this workflow in the manuscript to better explain the methodology used.

8. Please explain what is the ‘R statistic’. Is it Spearman correlation coefficient?

To clarify, we stated in the manuscript that the "R statistic" refers specifically to the ANOSIM (Analysis of Similarity) R statistic, which is used to quantify the degree of separation between groups based on distance matrices. In contrast, the Greek letter "ρ" (rho) represents the Spearman rank correlation coefficient.

9. The procedure leading to variable groups is unclear. Whas it arbitrary/subjective?

The procedure for grouping variables was based on ecological considerations, specifically the spatial scale and their relevance to forest structure. Variables were grouped according to how they correspond to different spatial scale of forest complexity from the landscape to the micro-habitat (layers). This approach was driven by the ecological significance of each variable in understanding forest structure and composition. We have clarified this process in the manuscript and improved Figure 3 to better explain the rationale behind these groupings.

10. Discussion is very elaborate and dissects each the main results. But, most of it does not derive from the MS results, so being arbitrary many times, as for instance, comments of flora diversity – when no flora was sampled, just ‘native/exotic’. S0, the discussion is absolutely exaggerated in its extension and mostly based on literature no the author´s own results. If not somewhat speculative or free-floating (not supported neither by results or literature). Another example of irrelevance is the whole discussion of several-scale analysis, from micro-habitat to stand scale. Nothing in the sampling procedure separates different spatial scales. I recommend that, in future versions of MS, the discussion to be much shorter and sticking to discussion of results and only comparisons with relevant similar results or needed to interpretation. Otherwise, suddenly readers get a new review article inside the MS.
I should remind that results are, in great extent, very trivial ones.
Therefore, I recommend Major Revision of the MS.

We have now provided a clearer explanation of our data collection process, including a more detailed description of how the variables were organized according to spatial scales. This is important because we structured our analysis with a hierarchical approach, where each level of scale (e.g., plot, understory, canopy) provided insights into how forest structure is influenced by different environmental and ecological factors. The primary goal of this study was not only to assess the structural complexity of two different woodland types on the Azores archipelago, but also to identify and analyze the drivers that differentiate these woodlands at multiple spatial scales. By organizing our analysis in this manner, we were able to tease apart the factors that influence forest structure, from local habitat features to broader landscape-level drivers.

We recognize that the results may appear highly descriptive, but this was by design. The complexity and scale of forest structure cannot be reduced to a single, straightforward conclusion. Instead, our goal was to understand the broader patterns and variability in forest structure across different scales and to explore the underlying factors responsible for these patterns. In doing so, we aimed to connect these findings to the broad historical and ecological context of the Azorean forests. This allowed us to discuss not only the structural differences between native and exotic woodlands, but also the possible drivers behind these differences, such as historical land-use, ecological succession, and other environmental factors that have shaped these forest types. Thus, while the analysis may seem descriptive, it provides a necessary foundation for understanding the dynamics of forest structure in the region, which is critical for future conservation and management strategies.
