# Supplementary material for: Landscape to microhabitat: Uncovering the multiscale complexity of native and exotic forests on Terceira Island (Azores, Portugal)
Source: PLoS One. 2025 Jun 16;20(6):e0326304. doi: 10.1371/journal.pone.0326304 (PMC12169593; doi:10.1371/journal.pone.0326304)
Supplement: Attachment — Submitted filename: Reviews comments V4.docx [file pone.0326304.s005.docx]

*I keep my comment on the title: The reader finds out that after all the study concerns one island out of nine islands, that needs to be addressed on the proposed title "Landscape to Microhabitat: Uncovering the Multiscale Complexity of Terceira Island forests (Azores, Portugal)". The authors have no data on the Azores just for 1/9 of the islands.*

We agree that the title should accurately reflect the geographic extent of the study, which is limited to Terceira Island. Accordingly, we have revised the title to:

**“Landscape to Microhabitat: Uncovering the Multiscale Complexity of** **Native and Exotic Forests on Terceira Island (Azores, Portugal)”**

While our study focuses exclusively on Terceira Island, we emphasize in the manuscript that the findings are likely applicable to other Azorean islands. This is because Terceira hosts the most representative and best-preserved remnants of several types of native forest, as well as typical secondary and exotic forest types found throughout the archipelago. Given this ecological representativeness, we consider Terceira an appropriate model for broader ecological inferences across the Azores. We have now clarified this point more explicitly in the manuscript to ensure that readers properly understand the study’s wider relevance and contextual framework.

*Abstract
By analysing landscape(…) we found that less accessible terrains are covered by the remnants of native forests. (…) plant species.
The authors are again stating that “they found” instead rephrase as:*

We have revised the sentence in the abstract to avoid the assertive phrasing “we found.” The revised sentence now reads:

**“Based on landscape, habitat, and microhabitat analyses, remnants of native forests appeared to be associated with less accessible terrains.”**

*The authors state: However, we recognize the limitation that precise historical land-use data are not available.
In fact historical data on the Azores do exist, it’s not of course digital cartography from the XV/XVI centuries but several descriptions, or recent publications that synthetise these ancient documents (see e.g. Dias, on the book: Açores e Madeira - A Floresta das Ilhas. Pp. 255-296. Público, Comunicação Social SA, Fundação Luso Americana para o Desenvolvimento e Liga para a Protecção da Natureza. ISBN: 978-989-619-103-0)*

We agree that while detailed historical cartography may not be available, valuable descriptive and synthesized sources do exist. In response, we now have revised the manuscript to include previously missing relevant references that discuss historical ecosystem distribution in the Azores.

*Abstract
Comment by the authors: While protected areas may be designated, their effective management and enforcement are essential to preserving native forests and controlling invasive species in exotic woodlands. In fact, we still see many areas in the archipelago (eg. In Flores, São Jorge and Faial) where the protection is only in the paper. Cattle is still moving around freely.
Reply: I understand the comment and the commitment but there cannot be implicit conclusions in a paper, or the results show what the authors state on the comment or my comments, based solely on the authors data, are still pertinent. (see final remark)
The statement “These insights emphasize (…) the preservation of native forests and the control of invasive species in exotic woodlands.” Is therefore hard to accept as it is, the authors data are not on the management, or protected versus unprotected areas, from the authors data these conclusions cannot be drawn.*

We agree that our study does not include quantitative data on the effectiveness of protected area management or enforcement in the Azores as it was not our objective. To address this, we have revised the statement to remain within the scope of our findings. The sentence now reads:

**“****These insights emphasize the importance of long-term monitoring and structural assessments in informing conservation efforts aimed at preserving native forests and managing invasive species in exotic woodlands.”**

While our field experience has exposed us to signs of inconsistent enforcement (e.g., cattle presence in some designated areas), such observations can be anecdotal and not sufficient to support empirical conclusions. We have therefore removed any implication that our data directly assess the effectiveness of protected area management.

*Introduction
The authors state:
However human activity significantly altered, with large areas being replaced by exotic species such as Cryptomeria japonica (L. f.) D.Don, 1841 and Pittosporum undulatum Vent., introduced for commercial forestry [29]. As a result, the Azores’s landscape has several patches of native and exotic woodlands in a matrix of agricultural fields [32,36], each with a different ecological importance. Native vegetation are crucial for maintaining the Azorean island’s biodiversity, acting as refuges for endemic plants and animals [37].
Delete “1841”*

The date “1841” has been removed from the species citation for *Cryptomeria japonica*, and the sentence has been corrected accordingly.

*Later in the comments the authors refer to 33 % for Pittosporum, 31 for % Cryptomeria, since both pastures, vineyards and many other human uses of the landscape are still missing I guess that “significantly altered” means close to extinction? The authors need here to introduce values/numbers on the % of native vegetation remains and native forest (and cite the sources).*

We have incorporated references that provide data on land-use changes and the current extent of native forest areas across the archipelago.

Specifically, we now cite Castanho et al. (2021), which offers a detailed analysis of land-use trends in the Azores between 1990 and 2018, and Triantis et al. (2010), which includes estimates of the remaining native forest cover and the historical context of habitat loss. These sources support the statement regarding significant and dramatic alteration of native vegetation, which refers not only to the replacement by *Cryptomeria japonica* and *Pittosporum undulatum* but also to broader land-use conversion for agriculture, pastures, and other human activities.

*Introduction ...consist primarily of broad-leaved evergreen trees. There are according to Elias (2016), Dias et al. (2021), and others several types of vegetation including some that are not broad-lead. To characterize the broad-leaved evergreen trees as such without any further hint or name is insufficient; include also the references: Dias, et al (2021). Vegetação dos Açores. In Capelo, J.; Aguiar, C. (Eds.), Vegetação de Portugal. Lisboa: INCM, p; Mucina, et al. "Vegetation of Europe: hierarchical floristic classification system of vascular plant, bryophyte, lichen, and algal communities". Applied Vegetation Science 19 (2016): 3-264; Fernández Prieto, et al. "Description of some new syntaxa from the Azores archipelago". International Journal of Geobotanical Research 2 1 (2012): 111-116.* [*http://dx.doi.org/10.5616/ijgr120007*](http://dx.doi.org/10.5616/ijgr120007) *The references included in my first review need to be included, there can be no cancellation on authors views or scientific approaches to vegetation study.*

Many thanks for remembering those references, we have now included the suggested sources—Dias et al. (2021), Mucina et al. (2016), and Fernández Prieto et al. (2012)—alongside the existing citations. Altogether, these references provide a global view of the diversity of vegetation types in the Azores, beyond the general characterisation of broad-leaved evergreen trees.

*The authors state: “each with a different ecological importance” this is unacceptable for island vegetation/plant diversity since none of the human introduced uses is ecological important, all being ecological destructive. The statement says a lot on authors views on conservation. It needs to be deleted.*

While we fully agree that native vegetation holds the highest ecological value and should be prioritized in conservation, we would like to clarify that our intent was not to equate the ecological importance of native and human-modified ecosystems, but rather to acknowledge the ecological roles that even altered landscapes can occasionally play.

To support this nuance, we refer to the study by Tsafack et al. (2021), which demonstrates that small patches of exotic forest—although human-modified—can serve as refuges for rare endemic arthropods. We have therefore revised the text to avoid any misinterpretation and to clarify that the ecological value of these systems is context-dependent and generally lower than that of native forests, but not necessarily negligible.

The revised sentence now reads:

**“As a result, the Azores’s landscape has several patches of native and exotic woodlands in a matrix of agricultural fields [32,42–44], each contributing differently to the current ecological dynamics of the island.”**

We hope this rephrasing addresses the concern while still reflecting the current complex ecological realities observed in the field.

*Introduction
Although the authors revised the use of “mosaic” this part of the introduction still needs to be improved using numerical data on types of cover.*

Following your previous comment, wee have enhanced the list of references in the Introduction. They now include sources that provide quantitative data and detailed descriptions of land-use types in the Azores. In particular, the study by Castanho et al. (2021) and Triantis et al (2010) offer comprehensive data on land-cover categories and their evolution over recent decades and has now been cited to support our characterization of the landscape mosaic. We chose not to duplicate these statistics directly in the Introduction, as such information can be found in the cited sources, allowing interested readers to consult the detail.

*Material and Methods.
1st paragraph
“…vegetation which were dominated” rephrase as “vegetation which are dominated”*

We have corrected the verb tense as recommended. The sentence now reads:
**“…vegetation which are dominated…”**

*What’s the meaning of “primarily consisting” means that are sites not dominated by Pittosporum?*

The expression “primarily consisting” was intended to indicate that the dominant woody species in the exotic forest plots is *Pittosporum undulatum*. To avoid ambiguity, we have revised the sentence to read:

**“The remaining nine sites were situated in areas dominated by the invasive species *Pittosporum undulatum*.”**

*Vegetation survey
“The objective was to obtain comprehensive data on the composition of forest species.” Rephrase objectives do not fit M&M*

We agree that objectives are generally not stated in the Materials and Methods section. However, this sentence refers specifically to the aim of the vegetation survey, not the overall study. To avoid confusion, we have rephrased the sentence for clarity and alignment with standard scientific writing:

**“****This survey aimed to collect detailed data on forest species composition across the study plots.”**

*Material and Methods.
Cryptomeria is not the dominate exotic species. Pittosporum dominated areas occupy 33% of forest surface in the Azores. Production forests (mostly, but not only (!), of Cryptomeria) occupy 31%. This is according to the «**State of the Environment Report of 2023» (Azorean Regional Government).
I accept that a further change on the title (apart from the geographical restriction) is not need, but use this data (numerical data) where required (see comment above)*

Following your previous comments, we confirm that this observation has been addressed in the revised manuscript.

*Material and Methods. Study Area and Site selection
The authors in their reply refer to a table, I could not find the reference in the text.*

The table referred to is included in the supplementary material, and we have now added an explicit reference to it in the main text to ensure clarity and accessibility for the reader.

*Material and Methods.
Although the authors have improved the text concerning plots and time, both the difference in size and the 10 years difference need to be included in the discussion.
Original comment: Even more taken into account that the authors state below that forests are homogeneous, were they 10 years ago? and then why should they still be homogeneous or even keep the same composition 10 years later?
I agree that the authors have the needed information, the question is where do readers get that information? And there is no reference to that in the text, it needs to be included with references preferably published in peer-reviewed publications (as stated above by the authors)
The authors further reply: Conducting new surveys could risk altering the forest structure due to trampling, which is particularly important since the focus of this study is on the structural complexity of the forests.
This seems to be a very poor, and not included, explanation.
The authors further reply: Similarly, (…) we believe the 10-year difference does not undermine this objective.
In my view this weakness on the methodological approach cannot be solved just by answering to the reviewers using private and unpublished information.
The authors have two choices to publish the information in the paper or to cite another source.*

We have added a paragraph in the Discussion section addressing the limitations related to the time gap and the difference in plot size between the native and exotic vegetation surveys. We acknowledge that these differences may introduce a degree of uncertainty, particularly in comparing composition over time.

Regarding the vegetation data for native plots, the surveys were conducted using a standardized protocol as part of earlier projects (ISLANDBIODIV and MOVECLIM), and are based on peer-reviewed methodology (as cited in Borges et al. 2018, Global Island Monitoring Scheme). Although these data are from 2012–2013, the sites are in well-preserved, minimally disturbed forest areas within protected areas. These forests are known for their structural stability, and currently no more recent peer-reviewed vegetation surveys exist for these exact plots.

Moreover, the wood species composition and abundance from those plots is already available in the supplementary material of the publication below, that we now cite in the methods section.

Borges, P.A.V., Cardoso, P., Fattorini, S., Rigal, F., Matthews, T.J., Di Biase, L., Amorim, I.R., Florencio, M., Borda-de-Água, L., Rego, C., Pereira, F., Nunes, R., Carvalho, R., Ferreira, M.T., Lopez, H., Pérez Delgado, A.J., Otto, R., Fernández Lugo, S., Nascimento, L. de, Caujapé-Castells, J., Casquet, J., Danflous, S., Fournel, J., Sadeyen, A.-M., Elias, R.B., Fernández-Palacios, J.M., Oromí, P., Thébaud, C., Strasberg, D. & Emerson, B.C. (2018). Community structure of woody plants on islands along a bioclimatic gradient. *Frontiers of Biogeography*, **10**(3-4): 1-31. DOI: 10.21425/F5FBG40295

As mentioned, new field sampling in these sensitive areas was avoided to prevent unnecessary disturbance to the forest structure, which is central to our study’s focus on structural complexity. We recognize this is a limitation and have now explicitly acknowledged it in the manuscript.

While we understand the reviewer’s concern, we believe that the combination of (i) the known stability of these forest systems, (ii) the location of plots in protected and undisturbed areas, and (iii) the use of a standardized and peer-reviewed vegetation survey protocol, provides a sufficient basis for using these data, while transparently communicating the limitation to readers.

*Material and Methods. Vegetation Survey
Original comment: The exotic forest plots were surveyed in the spring of 2023 within 20 x 20 (…). Why are the plots different in size?
Reply by the authors: The larger 50 x 50 meter plots for the native forests were chosen during the design of the ISLANDBIODIV project and we use the data gathered. The 20 x 20 meter plots in the exotic forests were selected based on forest height, accessibility and logistical considerations. Furthermore, the homogeneity of the plots supported the selection of this area to represent the forest structure being studied.
Homogeneity is a type of data, not shown in the paper, just assumed in the reply, if the authors can show or produce evidence of this then the reply should be transferred into the text. Otherwise, its complex to accept.*

To address our assumption on plot homogeneity, we have added the following sentence to the **Materials and Methods → Study Area and Site Selection** section, immediately after describing plot dimensions:

**“Prior to data collection, we conducted a brief field reconnaissance of each site. Although not formally quantified, this visual assessment confirmed that species composition and distribution were remarkably consistent across the entire area of each plot and its surroundings, supporting our use of differing plot sizes without introducing bias in structural complexity estimates.”**

We hope this addition clarifies our rationale and provides the transparency requested.

*Material and Methods. Vegetation Survey
Original comment: The protocol needs to be here described, but how can a protocol be applied in 2012 if it was published in 2018?
Authors reply: (…). The vegetation survey protocol was initially designed during earlier projects, notably ISLANDBIODIV and MOVECLIM, and was implemented in 2012 (…), leading to a delay in its official release.
I still don’t see the reply to “The protocol needs to be here described”
So, the readers are still missing a crucial point on methods used, describe the protocol even if just in a shorten version, was it adapted in any of its parts?*

A brief description of the vegetation survey protocol was indeed included in the original manuscript, beginning with the paragraph: *“Several important variables were measured during the surveys to assess the forest structure…”* This section outlines the variables recorded (e.g., species richness, basal area, and plant counts across strata), the plot setup (five-on-a-dice subplot arrangement), and the categorization of species and vegetation layers.

Following your previous comment, we have now made this section more explicit by introducing it with a clearer reference to the protocol, stating that it follows the standardized methodology developed through the ISLANDBIODIV and MOVECLIM projects and later published in GIMS paper from Borges et al. (2018).

*Material and Methods. Vegetation Survey
Original comment: Based on what did the authors segregate "introduced" and "invasive", a published list based on Azorean flora crossed with concepts like those published by Pyšek, Richardson and many others?
Authors reply: (…) The classification of species into "endemic," "native," "introduced," and "invasive" is based on Borges PAV, Costa A, (…). Principia, Parede. It compiles data from various sources and more recent updates available in AZORES BIOPORTAL
However, the original 2010 publication, for plants does not refer to naturalized versus invasive, just “Natu”, naturalized, please clarify this point, i.e. the distinction between introduced and invasive.*

While the classification of species as "endemic," "native," or "introduced" (“naturalized”) is based on the checklist by Borges et al. (2010) and continuously updated through the AZORES BIOPORTAL, the distinction between "introduced" and "invasive" species in our study specifically follows the legal framework established by Portuguese legislation—Decreto-Lei n.º 565/99, de 21 de Dezembro—which officially designates plant species considered invasive in the national territory, including the Azores.

We have now clarified this point in the Materials and Methods – Vegetation Survey section to ensure that our categorization criteria are transparent and supported by established sources. The revised text states:

**“****The classification of species as endemic, native, introduced, or invasive follows the regional checklist (Borges et al. 2010) and updated data from AZORES BIOPORTAL. Invasive status is further based on species listed in Decreto-Lei n.º 565/99, de 21 de Dezembro, which identifies legally recognized invasive plants in Portugal, including the Azores.”**

*Further comments:
On the results the authors state: “high level of pristine composition when considering the endemic tree species.”
This sentence seems to contradict the absence of data on pristine forests (see above), in fact it implies that there is a model of pristine vegetation, is there?*

To clarify, by “pristine composition,” we refer to forest areas where the vegetation is composed exclusively of endemic and native species, with no presence of introduced or invasive taxa. This definition aligns with current conservation usage in the Azores and is supported by the vegetation survey data provided in the supplementary material.

While we acknowledge that a fully undisturbed reference model for pristine forest is difficult to establish due to centuries of human influence and the recent spread of the invasive plant *Hedychium gardnerianum* (Ginger lily), the native forest plots included in our study were selected specifically for their high naturalness and minimal anthropogenic disturbance, as documented in earlier surveys. The several works on arthropod surveys and monitoring on those plots cited in this manuscript confirm the naturalness of those plots, in which endemic arthropods dominate in the community.

*Discussion
Is lacking a thorough discussion of possible drawbacks of methodological problems and adaptations, and in my view is clearly over-extended.*

In response to earlier comments, we have now added a dedicated paragraph in the Discussion section that directly addresses methodological limitations, including the differences in plot size and the 10-year gap between vegetation surveys. This paragraph also discusses the ecological stability of native forests compared to the dynamic nature of exotic ones, and the rationale for using existing data to avoid additional disturbance in sensitive areas.

*1st paragraph: “Furthermore, (…) invasion patterns” it seems a circular though/statement, if invaded i.e. Pittosporum dominated sites have a distinct structure then structure is an indicator of the dominance of Pittosporum….why should anyone use structure if the “indicator” is at plain sight?*

Our study focused on two well-defined forest types—native and *Pittosporum*-dominated exotic forests—to clearly characterize the structural and compositional differences between these extremes. While it is true that in such clear-cut cases, the dominance of *Pittosporum* or native species may be visually evident, the purpose of analyzing forest structure was not to detect what is already apparent, but to understand how structural metrics reflect underlying ecological processes and to assess their potential utility in more ambiguous or transitional forest areas.

Indeed, in many parts of the Azores, forest patches exhibit mixed compositions or early stages of invasion, where dominant species may not be immediately obvious or where structural indicators could provide early signs of disturbance or ecological change. Although developing a structural indicator for such transitional states was not the goal of this study, our results lay the groundwork for future applications where structure-based assessment could support forest monitoring and management beyond visually evident cases.

*1st Paragraph “The findings of this study highlight the necessity of examining forest structure at various spatial scales, as the differentiation between native and exotic forests exhibited significant variation depending on the spatial level under consideration.”
Consider discussing your results on a multi-invasion process, i.e. where Pittosporum is the dominant invading phanerophyte but other invading plants at distinct forest strata are also paving their way into the landscape.*

We agree that a multi-invasion perspective—considering the stratified nature of invasion across different forest layers—is highly relevant and supported by our results. In response, we have expanded the discussion to acknowledge that while *Pittosporum undulatum* is the dominant invasive phanerophyte in the canopy, our data also show the presence and increasing influence of other invasive and introduced species in the understory and ground layers.

This vertical stratification of invasion reflects a multi-invasion process, where different non-native species establish and spread within distinct forest strata, likely driven by varying ecological niches, dispersal strategies, and competitive dynamics. This layered invasion may act synergistically, facilitating further structural and compositional transformation of the forest ecosystem over time. We have now included this interpretation in the discussion to highlight the broader ecological implications of our findings.

*2nd Paragraph: “concerning for the delicate ecosystem of the Azores” well very threatened, reduced, relictual?*

We agree that “delicate” may be too vague and does not fully convey the urgency and vulnerability of the Azorean native forest ecosystem. In response, we have revised the sentence to remove this term.

*4th Paragraph: “human presence on Terceira Island has led to the intentional and unintentional introduction of non-native species, particularly in forests accessible for resource extraction and agriculture.” If this this is a discussion, please insert a citation for this statement.*

We have now added appropriate citation to support the statement regarding the intentional and unintentional introduction of non-native species associated with human activity on Terceira Island.

*5th Paragraph: “The enhanced accessibility (...) replacement of indigenous species with exotic ones” replacement would be understood by most of the readers as a direct replacement where one species (invasive) outcompeted a native/endemic. This was not the case. In the Azores vegetation was destroyed and used and later invaded, invasive species were introduced mostly in “empty” landscapes.*

We agree that the historical transformation of Azorean vegetation was primarily driven by habitat destruction and land conversion, with invasive species often colonizing these altered or “empty” landscapes. This is indeed reflected in several of the publications cited throughout the manuscript.

However, our use of the term “replacement” was intended to refer not only to historical processes, but also to **ongoing impacts**, where exotic and invasive species continue to expand and exert pressure on remnant native communities. To avoid confusion, we have revised the wording in the manuscript to clarify this distinction and better reflect the sequence of landscape use, abandonment, and subsequent invasion.

The revised sentence now reads:

**“****The enhanced accessibility has facilitated the widespread transformation of landscapes, where indigenous species were initially displaced through land-use change, and later further impacted by the expansion of exotic and invasive species into these disturbed areas.”**

*5th Paragraph: “By contrast, exotic forests, which have been established in more easy-to-access areas, exhibit spatial traits that align with historical human activity and land-use change.” Easy to access areas were more easily used by humans (agriculture etc) and therefore invaded because they were empty…*

As noted in our previous response, we have revised the relevant sentence to better reflect the historical sequence of land-use conversion and subsequent invasion. The updated version now clarifies that easy-to-access areas were initially used for human activities such as agriculture, and later became susceptible to invasion following abandonment.

*On the “The canopy ...”
Complexity is here assumed in a static view but if native forest is older (even very optimistically stated as close to pristine…) and exotic younger then heterogeneity would be expected do to time alone, i.e. the exotic forests are still far from “mature” where “natives” are mature forests. Please discuss.*

We agree that the age difference between native and exotic forests is an important factor that may influence their structural characteristics. Native forests in the Azores, particularly those included in this study, are relatively older, less disturbed, and closer to mature or late-successional stages, which contributes to their structural and compositional homogeneity. In contrast, exotic forests, especially those dominated by *Pittosporum undulatum*, are more recent in origin and may not have reached a stable or mature structural state.

We have now added a brief discussion acknowledging that part of the observed structural heterogeneity in exotic forests could be attributed to their relative youth and ongoing successional dynamics. However, we also note that these forests are composed of fast-growing, invasive species selected for their capacity to rapidly colonize and dominate disturbed environments. As such, the structural heterogeneity observed is likely not just a function of age, but also of species traits and the legacy of land-use history.

We have added the following sentence to the Discussion:

**“It is important to consider that the observed structural heterogeneity in exotic forests may partly reflect their younger age and earlier successional stage relative to native forests. However, given their dominance by fast-growing invasive species and history of colonizing previously disturbed areas, this heterogeneity also reflects ongoing ecological dynamics that may not necessarily lead to convergence with native forest structure over time.”**

*On the “temporal dynamics”
“The findings indicate a historical trajectory (...) from pristine native forest ecosystems to increasingly disturbed exotic stands.” This not the scope of this publication that does not include historical data, even less on pristine forest. Delete the sentence.*

As suggested, we have removed the sentence referring to a historical trajectory from pristine native forest to disturbed exotic stands, as this interpretation was beyond the scope of the data presented in this study. The entire paragraph has been deleted accordingly.

*“Conservation efforts should prioritize protecting less accessible areas where native species have a competitive advantage. Conversely, restoration in exotic forests should aim to enhance structural resilience by controlling invasive species, particularly in the ground and canopy layers, while promoting native species to improve overall habitat quality”.
Results prove clearly that conservation efforts should prioritize invaded areas, promoting their conversion to a higher degree of “nativeness” and not to preserve what is already protected as stated by the authors as close to pristine.*

We agree that restoration of invaded areas is essential for increasing ecosystem resilience and promoting native biodiversity. However, we respectfully disagree with the implication that conservation should focus solely on converting exotic forests, rather than also maintaining and reinforcing protection of existing native forest remnants.

Even though some native forest areas are legally protected and may appear close to “pristine,” they remain highly vulnerable due to ongoing external pressures such as edge effects, invasive species encroachment, climate change, and limited enforcement of protection measures. In our view, conservation efforts must be holistic and include both the **active restoration** of degraded areas and the **continued safeguarding and management** of native habitats—particularly those located in less accessible areas that naturally limit human disturbance.

Additionally, our use of the term “conservation actions” includes more than direct field interventions. It also encompasses efforts such as public awareness, education, and monitoring—crucial elements for long-term success in island biodiversity conservation as we cannot protect what we are ignoring.
